# The Anterior Chamber Depth and Retinal Nerve Fiber Layer Thickness in Children

**DOI:** 10.1155/2014/538283

**Published:** 2014-11-09

**Authors:** Jacky W. Y. Lee, Gordon S. K. Yau, Tiffany T. Y. Woo, Doris W. F. Yick, Victor T. Y. Tam, Can Y. F. Yuen

**Affiliations:** Department of Ophthalmology, Caritas Medical Centre, 111 Wing Hong Street, Kowloon, Hong Kong

## Abstract

*Purpose*. To investigate the correlation of anterior chamber depth (ACD) with the peripapillary retinal nerve fiber layer (RNFL) thickness, age, axial length (AL), and spherical equivalent in children. *Subjects*. Consecutive subjects aged 4 to 18 were recruited. Visually disabling eye conditions were excluded. Only the right eye was included for analysis. The ACD was correlated with RNFL thickness, age, spherical equivalent, and AL for all subjects. Subjects were then divided into 3 groups based on their postcycloplegic spherical equivalent: myopes (<−1.0 D), emmetropes (≥−1.0 to ≤+1.0 D), and hyperopes (>+1.0 D). The ACD was compared among the 3 groups before and after age adjustment. *Results*. In 200 subjects (mean age 7.6 ± 3.3 years), a deeper ACD was correlated with thinner global RNFL (*r* = −0.2, *r* ^2^ = 0.06, *P* = 0.0007), older age (*r* = 0.4, *r* ^2^ = 0.1, *P* < 0.0001), myopic spherical equivalent (*r* = −0.3, *r* ^2^ = 0.09, *P* < 0.0001), and longer AL (*r* = 0.5, *r* ^2^ = 0.2, *P* < 0.0001). The ACD was deepest in myopes (3.5 ± 0.4 mm, *n* = 67), followed by emmetropes (3.4 ± 0.3, *n* = 60) and then hyperopes (3.3 ± 0.2, *n* = 73) (all *P* < 0.0001). After age adjustment, myopes had a deeper ACD than the other 2 groups (all *P* < 0.0001). *Conclusions*. In children, a deeper ACD was associated with thinner RNFL thickness, older age, more myopic spherical equivalent, and longer AL. Myopes had a deeper ACD than emmetropes and hyperopes.

## 1. Introduction

The anterior chamber depth (ACD) is an important biometrical parameter because variations of ACD in adults have been associated with different glaucoma subtypes. A deeper ACD has been found in those with pigment dispersion syndrome [1] while a shallower ACD has been associated with a spectrum of angle closure conditions including primary angle closure suspect, primary angle closure glaucoma, and acute angle closure [2–4]. The ACD has also been found to vary depending on ethnicity, with smaller anterior segment dimensions in Asian compared to Caucasian adults [5, 6]. The ACD is dynamic during childhood with the most rapid change occurring between the ages of 6 and 10 [7]. Understanding the associations of ACD with childhood helps us to determine the historical factors that may influence the anterior segment dimensions in adulthood. Few studies have reported the relationship between ACD and peripapillary retinal nerve fiber layer thickness (RNFL) in children. The purpose of this study was to investigate the association of ACD with RNFL, age, axial length, and spherical equivalent in a population of Chinese children.

## 2. Patients and Methods

The study was conducted in accordance with the Declaration of Helsinki and no patient personal data was disclosed in the study. Study approval was obtained from the Institutional Review Board of the Hospital Authority of Hong Kong. Informed consent was obtained from the parents or legal guardian of the subjects. The authors declare no financial or proprietary interests.

This cross-sectional study recruited consecutive pediatric subjects aged 4 to 18, attending the ophthalmology specialist outpatient clinic of Caritas Medical Centre in Hong Kong Special Administrative Region, China, from 2013 to 2014. Subjects with only one eye, ocular tumors, congenital glaucoma, congenital cataract, congenital nystagmus, microphthalmos, optic nerve or retinal disease, active cornea infections, corneal scars, and severe visual impairment of any cause (Snellen best corrected visual acuity ≤ 0.1) were excluded.

All subjects underwent a complete ophthalmological examination including ocular alignment and motility assessments as well as anterior and posterior segment examinations after pupil dilation with a tropicamide 1% and phenylephrine hydrochloride 2.5% ophthalmic solution (Mydrin-P; Santen Pharmaceutical, Osaka, Japan). Cycloplegia for refraction was achieved with cyclopentolate hydrochloride 1% (Bausch & Lomb, Rochester, NY, USA).

### 2.1. Spherical Equivalent, Axial Length, and ACD

All subjects had cycloplegic refraction with 3 drops of cyclopentolate hydrochloride 1% (Bausch & Lomb, Rochester, NY, USA) administered 5 minutes apart to relieve all accommodative component. After at least 30 minutes, postcycloplegic autorefraction with a kerato-refractometer (Topcon KR-8900 by Topcon Europe Medical B.V., Capelle a/d Ijssel, The Netherlands) was performed by an optometrist with at least 5 years of experience with pediatric assessment. The spherical equivalent was calculated in diopters (D). Axial length and ACD measurements in millimeters (mm) were obtained with the noncontact optical biometry (IOLMaster; Carl Zeiss Meditec AG, Berlin, Germany). Axial length was taken as the distance between the anterior corneal vertex and the retinal pigment epithelium along visual fixation after automatic adjustment for the RNFL thickness. A reliable axial length measurement consisted of a signal-to-noise ratio ≥ 2.0. The ACD was taken as the distance between the anterior corneal surface and the anterior lens surface; a minimum of 5 readings was taken.

### 2.2. Peripapillary RNFL Thickness

The Spectralis Spectral-Domain Optical Coherence Tomography (OCT, Heidelberg Engineering, Carlsbad, CA, USA) was performed after cycloplegia, by a single, imaging technician who was masked to subjects' clinical information. Scans were centred on the optic disc with a scanning diameter of 3.5 mm and 768 A-scans were obtained using the high speed (HS) mode. To improve image quality, automatic real time (ART) function was used to obtain multiple frames during scanning and to optimize images by noise reduction. Scans were repeated 3 times and assessed for signal strength and centration, and the best quality scan was selected. Scans with signal strength quality ≤ 16 or poor centration were excluded. RNFL thickness was analysed with the RNFL Single Exam Report OU with fovea-to-disc technology. The RNFL thickness of each of the 4 quadrants and the global RNFL thickness were recorded in micrometers (*μ*m).

### 2.3. Statistics

Subjects were divided into 3 groups based on their postcycloplegic spherical equivalent: myopic (< −1.0 D), emmetropic (≥ −1.0 to ≤ +1.0 D), and hyperopic (> +1.0 D). Only the right eye of each subject was used for statistical analysis. Statistical significance was considered when *P* < 0.05. Means were expressed with standard deviations.

The following were compared using one-way ANOVA with Tukey's multiple comparison test for the 3 spherical equivalent groups:

- anterior chamber depth (ACD) both before and after age adjustment,
- age.

Pearson correlation was used to analyze the association between the following parameters:

- ACD versus RNFL (global and quadrant) thicknesses,
- ACD versus age,
- ACD versus axial length,
- ACD versus spherical equivalent,
- ACD versus global RNFL thickness in the myopia, emmetropia, and hypermetropia groups.

Linear regression analysis was used to analyze the association between the following parameters:

- ACD versus global RNFL thicknesses,
- ACD versus age,
- ACD versus axial length,
- ACD versus spherical equivalent.

## 3. Results

The mean age of 200 subjects was 7.6 ± 3.3 years. There were 97 female and 103 male subjects, all of Chinese ethnicity. The ACD was deeper in boys (3.4 ± 0.4 mm) than in girls (3.3 ± 0.3 mm) (*P* = 0.008). There were 67 (33.5%) myopic eyes, 60 (30.0%) emmetropic eyes, and 73 (36.5%) hyperopic eyes. The mean RNFL thickness in each of the 4 quadrants and the global RNFL thickness have been summarized in Table 1. There were significant and negative correlations between the ACD and the inferior (*r* = −0.2, *P* = 0.001) and global (*r* = −0.2, *P* = 0.0007) RNFL thickness. There was no significant correlation between the ACD and the superior, nasal, or temporal RNFL (all *P* ≥ 0.06) (Table 1).

**Figure 1:**
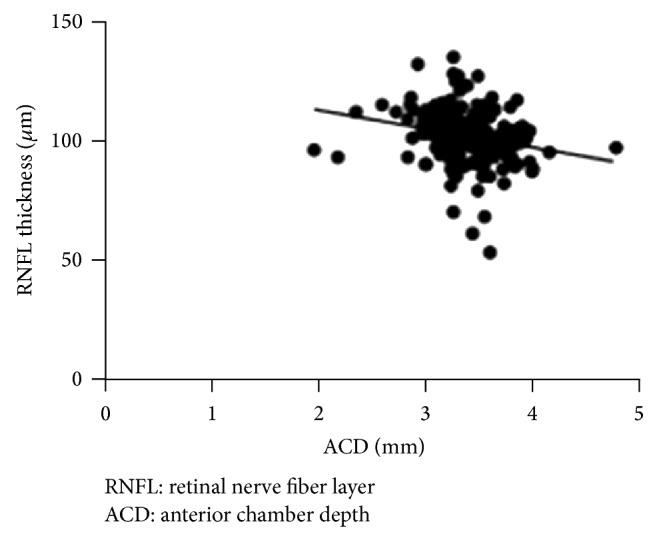
Linear regression analysis between global RNFL thickness and ACD.

The global RNFL (*r* = −0.2, *P* = 0.0007) and spherical equivalent (*r* = −0.3, *P* < 0.0001) were negatively correlated with ACD. Age (*r* = 0.4, *P* < 0.0001) and axial length (*r* = 0.5, *P* < 0.0001) were positively correlated with ACD. There was a negative and linear correlation between the global RNFL (*r* ^2^ = 0.06, *P* = 0.0007) and the spherical equivalent (*r* ^2^ = 0.09, *P* < 0.0001) with ACD. There was a positive and linear correlation between age (*r* ^2^ = 0.1, *P* < 0.0001) and axial length (*r* ^2^ = 0.2, *P* < 0.0001) with ACD (Table 2, Figure 1).

The mean spherical equivalent was statistically different among the myopic (−3.8 ± 2.2 D), emmetropic (+0.05 ± 0.5 D), and hyperopic groups (+3.0 ± 1.6 D) (all *P* < 0.0001). The ACD was deeper in the myopic group (3.5 ± 0.4 mm) than in the emmetropia (3.4 ± 0.3 mm) and hyperopic (3.3 ± 0.2 mm) groups (both *P* < 0.0001); there was no significant difference in ACD between the emmetropic and hyperopic groups (*P* > 0.05). The myopic group (9.6 ± 3.9 years) was older than the emmetropic (6.8 ± 2.8 years) and hyperopic groups (6.5 ± 1.9 years) (both *P* < 0.0001). When adjusted for age, the ACD was still significantly deeper in the myopic group than in the emmetropic and hyperopic groups (both *P* < 0.0001), again without significant difference between the emmetropic or hyperopic group (*P* > 0.05). When subdivided by spherical equivalent and adjusted for age, only the ACD in the emmetropic group demonstrated a negative correlation with the global RNFL (*r* = −0.4, *P* = 0.01). The above results have been summarized in Table 3.

## 4. Discussion

In contrast to adults in whom the ACD becomes more shallow with age [8], the ACD in our pediatric population (aged 4 to 18) became deeper with advancing age (*r* = 0.4, *r* ^2^ = 0.1, *P* < 0.0001). Our findings were consistent with those of Breslin et al. and Wong et al. who also reported a continued deepening of the ACD in the growing child and that the process eventually slowed down after the age of 10 to 11 [7, 9]. In our study, boys had a significantly deeper ACD than girls (3.4 ± 0.4 mm versus 3.3 ± 0.3 mm, *P* = 0.008), which was compatible with the findings of a previous Australian study that also reported a deeper ACD in boys [10]. It has been reported that adult females in Singapore have 43% more risk of developing angle closure than their male counterparts [11] and from the findings of our study, it seems that this gender difference in ACD may be predetermined from childhood.

The inferior RNFL (130.3 ± 21.2 *μ*m) was the thinnest and the nasal RNFL (63.6 ± 16.4 *μ*m) was the thinnest of the 4 quadrants. These findings were in good agreement with a recent population study by Bueno-Gimeno et al. reporting that the inferior RNFL (130.11 ± 21.33 *μ*m) was the thickest while the nasal RNFL (72.12 ± 16.76 *μ*m) was the thinnest in children aged 6 to 17 years [12]. A deeper ACD was associated with a thinner inferior and global RNFL thickness, with the global RNFL thickness showing more significant correlation (*r* = 0.2, *r* ^2^ = 0.06, *P* = 0.0007), although the correlations were weak. There were no significant associations between ACD and the other RNFL quadrants (all *P* ≥ 0.06). When separated into different refractive groups and adjusted for age, the negative correlation between the ACD and global RNFL was only apparent in the emmetropic group. To the best of our knowledge, this is the first study analysing the association between ACD and OCT measured RNFL thickness in a pediatric population.

Our study is also one of the first to report the differences in ACD among different refractive groups (myopia, emmetropia, and hyperopia) within the same population in Asian children. After adjustments for age, we found that myopic children had a deeper ACD than their emmetropic and hyperopic counterparts (both *P* < 0.0001) while there was no significant difference in ACD between emmetropic and hyperopic children (*P* > 0.05). Our findings were compatible with a Polish study that examined 86 children (aged 4 to 17) and found that the ACD was deeper in myopic eyes (3.18 ± 0.31 mm), followed by emmetropic (2.92 ± 0.11 mm) and hyperopic eyes (2.73 ± 0.2 mm) (*P* < 0.01) [13]. However, the overall ACD range (2.92 to 3.18 mm) in the Polish population was shallower than in our population that had a similar age distribution (ACD range: 3.3 to 3.5 mm). This difference could be attributed to a greater myopic shift in East Asian children [14], since a more myopic spherical equivalent was correlated with a deeper ACD in both our studies [13]. Furthermore, when compared to a population of Tibetan children aged 6 to 16, their ACD range (3.38 to 3.61 mm) was of a closer resemblance to our population [15].

Our study found a significant correlation between a deeper ACD and a more myopic spherical equivalent (*r* = −0.3, *r* ^2^ = 0.09, *P* < 0.0001) and a longer axial length (*r* = 0.5, *r* ^2^ = 0.2, *P* < 0.0001). These results were consistent with a French study reporting a deeper ACD following axial length elongation and myopia [16]. Likewise, Garner et al. found that the rate of ACD deepening was significantly greater by 0.012 mm/year (*P* = 0.014) in myopic (≤−0.5 D) compared to nonmyopic children in Nepal [17]. However, there is no consensus in the literature regarding the association between ACD and myopia in children. The rate of myopic progression was not correlated with ACD in a study on Singaporean children [18]. Similarly, a Taiwanese study involving children aged 6 to 13 found that myopia was not correlated with ACD [19]. Furthermore, a study on Northern Ireland children reported an absence of correlation between ACD and spherical equivalent refraction [7]. These differences could potentially be related to variations of the spherical equivalent range among different geographical regions as the differences in myopic progression were 3 times more in a Singaporean versus a Northern Ireland pediatric population of similar baseline age [7, 20]. On the other hand, these differences cannot be fully explained by differences in ethnicity alone since a study by Logan et al. found no significant difference in the ACD among children of Asian, Caucasian, or African ethnicity [21]. In adulthood, the ACD in the Asian population is considerably shallower than the Caucasian population [5, 6]. Based on findings in the pediatric population showing a similar or even deeper ACD in Asian children (secondary to a greater myopic shift), the shallower ACD in Asian adults is likely attributed to the greater anterior expansion of the lens and posterior segment structures that occur primarily in adulthood, predisposing Asian adults (especially females) to a greater risk of angle closure diseases particularly between the age of 55 and 70 [22].

Our study had its limitations. Firstly, this was only a cross-sectional study and did not provide information on the serial changes that occur in the ACD during childhood. Secondly, we only analyzed the postcycloplegic ACD in all subjects. A previous study found that the ACD could be increased by 0.05 to 0.06 mm after cycloplegia [23] but as our population consisted of children aged 4 to 18, the accommodative power would vary considerably; thus, the authors decided that using a standardized postcycloplegic ACD measurement in all subjects would bring more consistency to our comparisons. Thirdly, the association between ACD and the inferior and global RNFL was only a weak correlation, and larger scale studies are warranted to establish the clinical significance of such association.

In Chinese adults, a shallower ACD comes with age which in turn increases the risk for angle closure glaucomas, which may in turn decrease the RNFL thickness. But in children, the opposite is true: a deeper ACD was associated with a thinner inferior and global OCT-measured peripapillary RNFL. A deeper ACD was also correlated with advancing age, myopia, and axial length elongation. When adjusted for age, myopes had a deeper ACD than both emmetropes and hyperopes who had similar ACD.

## Figures and Tables

**Table 1:** Pearson correlation between anterior chamber depth and the global and quadrant retinal nerve fiber layer.

	Inferior	Superior	Nasal	Temporal	Global
Mean RNFL thickness (*μ*m)	130.3	125.9	63.6	87.6	102.0
Standard deviation (*μ*m)	21.2	20.6	16.4	19.3	11.0
Pearson *r*	−0.2	−0.1	−0.1	−0.05	−0.2
*P* value	0.001^*^	0.06	0.1	0.5	0.0007^*^

^*^Statistically significant.

**Table 2:** Correlation between anterior chamber depth and global retinal nerve fiber layer, age, axial length, and spherical equivalent.

	Pearson correlation		Linear regression		
	Pearson *r*	*P* value	*r* ^2^	Slope	*P* value
ACD versus global RNFL	−0.2	0.0007^*^	0.06	−7.9 ± 2.2	0.0007^*^
ACD versus age	0.4	<0.0001^*^	0.1	3.7 ± 0.7	<0.0001^*^
ACD versus axial length	0.5	<0.0001^*^	0.2	2.0 ± 0.3	<0.0001^*^
ACD versus spherical equivalent	−0.3	<0.0001^*^	0.09	−2.0 ± 0.7	<0.0001^*^

^*^Statistically significant.

ACD: anterior chamber depth.

RNFL: retinal nerve fiber layer.

**Table 3:** Differences in age, spherical equivalent, axial length, and anterior chamber depth in myopic, emmetropic, and hyperopic children.

	Myopic group (*n* = 67)	Emmetropic group (*n* = 60)	Hyperopic group (*n* = 73)	Statistical significance
Age (years)	9.6 ± 3.9^a^	6.8 ± 2.8^b^	6.5 ± 1.9^c^	*P* < 0.0001^*^ for a versus b/c *P* > 0.5 for b versus c
Sex (M : F)	36 : 31	37 : 23	30 : 43	N/A
Spherical equivalent (D)	−3.8 ± 2.2	+0.05 ± 0.5	+3.0 ± 1.6	*P* < 0.0001^*^ between all groups
ACD (mm)	3.5 ± 0.4^a^	3.4 ± 0.3^b^	3.3 ± 0.2^c^	*P* < 0.0001^*^ for a versus b/c *P* > 0.5 for b versus c
ACD (mm) after age adjustment	3.5 ± 0.4^a^ (*n* = 35)	3.3 ± 0.3^b^ (*n* = 35)	3.3 ± 0.2^c^ (*n* = 35)	*P* < 0.0001^*^ for a versus b/c *P* > 0.5 for b versus c
Correlation between ACD and global RNFL after age adjustment	Pearson *r* = 0.03 (*P* = 0.8)	Pearson *r* = −0.4 (*P* = 0.01)^*^	Pearson *r* = −0.05 (*P* = 0.8)	N/A

^*^Statistically significant.

The superscripts “a, b, and c” are used to demonstrate the statistical significance between the groups.
